# Acute Myocarditis From the Use of Selective Androgen Receptor Modulator (SARM) RAD-140 (Testolone)

**DOI:** 10.7759/cureus.21663

**Published:** 2022-01-27

**Authors:** Rana Prathap Padappayil, Arundhati Chandini Arjun, Jonathan Vivar Acosta, Wael Ghali, Mohsin Sheraz Mughal

**Affiliations:** 1 Internal Medicine, Monmouth Medical Center, Long Branch, USA

**Keywords:** pulmonary edema, selective androgen receptor modulator, myocarditis, shortness of breath, acute heart failure

## Abstract

Selective Androgen Receptor Modulators (SARMs) work at the level of the androgen receptor and are potential alternatives to testosterone supplementation in patients with hypogonadism. We report the case of a young male who presented with possible acute myocarditis from self-medication with SARM for bodybuilding.

## Introduction

The clinical presentation of myocarditis can vary from insidious self-limiting chest pain to overt heart failure, cardiogenic shock, and even death. The etiology is multifold and could be either infectious (viral, bacterial, and protozoal), or non-infectious (cardiotoxins, chemotherapy medications, or other systemic illnesses) [1]. Myocarditis is defined to be acute if the symptoms develop in less than three months from the inciting event. Acute myocarditis has a prevalence of 10-22 cases per 100,000 persons annually. For a disease process with a wide range of possible etiologies, in patients with myocarditis, sometimes an identifiable etiology is missing, and hence, myocarditis is frequently idiopathic.

Selective Androgen Receptor Modulators (SARMs) are medications that work at the androgen receptor and function as either an agonist or an antagonist [2]. Testosterone supplementation is usually the mainstay of therapy in patients with hypogonadism. However, testosterone is often aromatized to estrogen in the body, leading to gynecomastia, and has a rather unfavorable side effect profile, including male pattern baldness, erythrocytosis, alterations in spermatogenesis, and dyslipidemia to name a few. SARMs have been touted as a safer alternative due to their selective action at the androgen receptor level [3].

We present the case of a young healthy male patient who presented with complaints of shortness of breath after self-medicating himself with SARM RAD-140 (testolone) for performance enhancement and muscle building. A presumptive diagnosis of SARM RAD-140-induced acute myocarditis was made.

## Case presentation

The patient is a 32-year-old male who presented with complaints of shortness of breath on exertion that started one day prior. He has a medical history of Type I Diabetes Mellitus, which is currently controlled on home insulin injections. He uses insulin glargine 45 units daily at bedtime and insulin lispro 18 units three times a day with meals. His last hemoglobin A1c (HbA1c) prior to the hospitalization was 10.2 mmol/mol. He reports no long-term complications of diabetes, including neuropathy, nephropathy, or retinopathy. He never had the COVID-19 infection and has not been vaccinated. He also has a history of inhaled cocaine and injected heroin use in the past, both of which he has quit for the last two years. He is currently on medication-assisted treatment for opioid use with buprenorphine 8 milligrams sublingually three times a day and is reported to be compliant with the same. The patient also recently started taking a SARM RAD-140 (testolone), which he purchased online. He has been using them for performance enhancement and for increasing his muscle mass. His last use of testolone was a day before the hospitalization.

According to the patient, he was in his usual state of health one day before admission when he noticed that he had to stop to catch his breath while climbing a flight of stairs to his apartment. This is something that he can usually do without being short of breath, and this concerned him. Since then, he became increasingly short of breath to the point that he couldn’t even walk to the bathroom. This prompted a visit to the emergency department. He denied chest pain or tightness, cough, palpitations, orthopnea, recent respiratory illnesses, or similar episodes in the past.

The patient was awake, alert, oriented, and in mild respiratory distress. He was febrile with a temperature of 99.9 °F, heart rate 115 beats per minute, blood pressure 126/86 mm Hg, respiratory rate of 27 breaths per minute, and oxygen saturation was 93% on 2 liters of oxygen supplementation via nasal cannula. Physical examination was unremarkable except for fine crackles at the bases of bilateral lung fields. The jugular venous pulsations were not elevated, and pedal edema was not apparent. Laboratory values were significant for an elevated troponin I level of 77.1 ng/ml, B-type natriuretic peptide level of 1288 pg/ml, erythrocyte sedimentation rate of 75 mm per hour, and a C-reactive protein level of 147.22 mg/L (Table 1).

**Table 1:** Laboratory tests and values on admission (Hospital Day 1) WBC: white blood cells; CK-MB: creatine kinase-MB

Laboratory Test	Value	Unit	Normal Range
WBC Count	7.9	K/CMM	4.5-11.0
Erythrocyte Sedimentation Rate	75	mm/hr	0-15
Ferritin	173.7	ng/mL	10-322
Creatinine Kinase	559	IU/L	20-190
CK-MB fraction	9.2	%	0-1.4
B-type natriuretic peptide	1288	pg/mL	0-100
C-reactive protein	147.22	mg/L	0-7.00
Troponin I	77.11	ng/mL	0.04-0.80
D-Dimer	1.57	FEU	0-0.5

**Figure 1:**
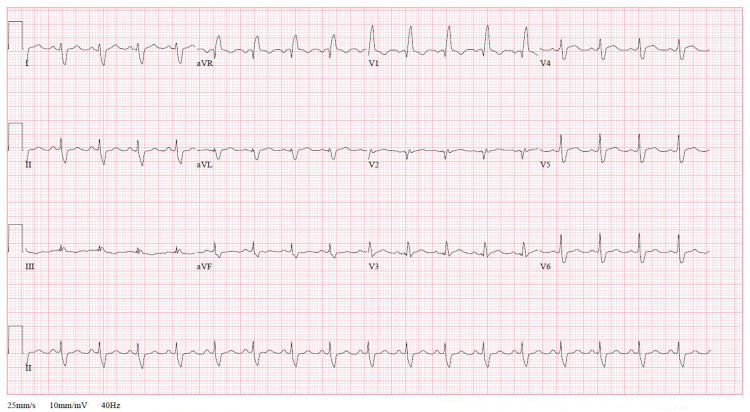
Electrocardiogram (EKG) on admission (Hospital Day 1) revealed sinus tachycardia and a right bundle branch block

A procalcitonin level and three COVID-19 tests via polymerase chain reaction (PCR) were negative. An upper respiratory pathogen panel via PCR from a nasopharyngeal swab was negative for identifiable viral infections. An antibody panel for viral infections demonstrated elevated IgG antibody levels against Epstein-Barr Virus (EBV) nuclear antigen, and cytomegalovirus (CMV) suggestive of past infections. A urine toxicology screening and serum toxicology test via liquid chromatographic method were negative. Electrocardiogram (EKG) on admission was significant for sinus tachycardia and a right bundle branch block. No prior EKGs were available for comparison (Figure 1).

**Figure 2:**
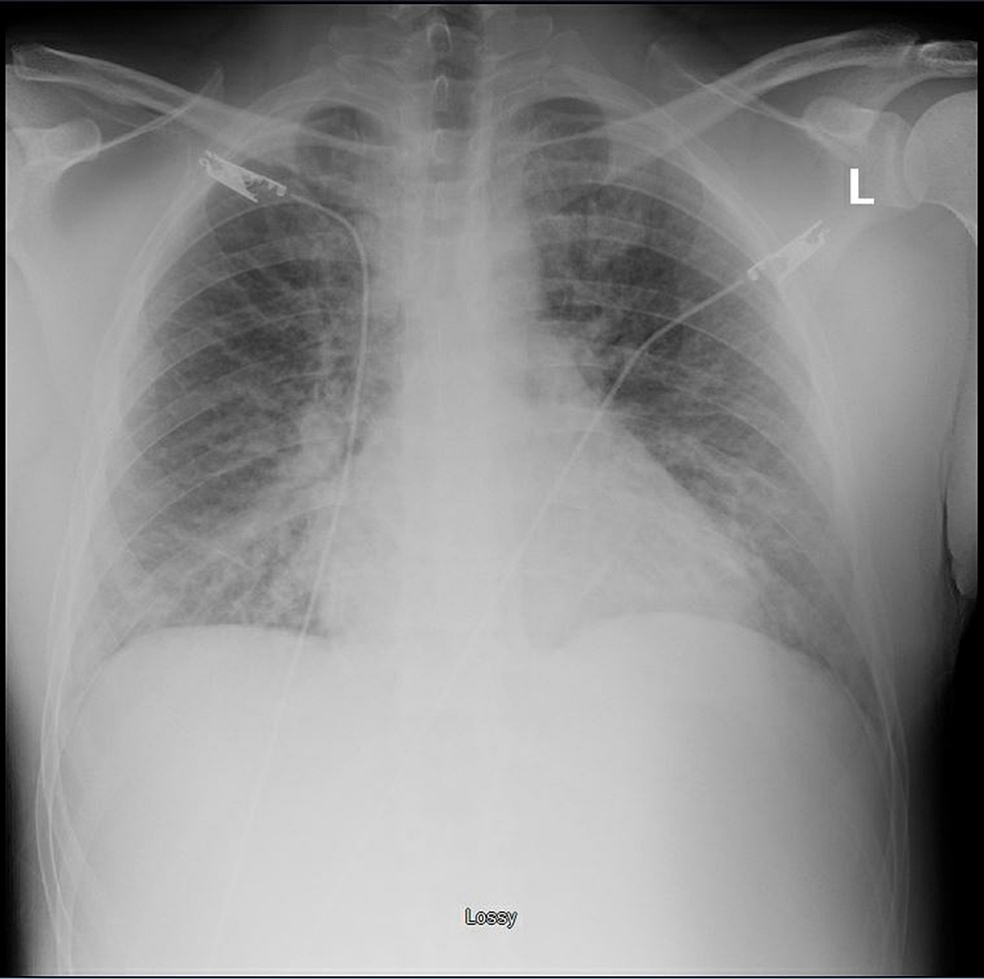
A Chest X-ray on admission (Hospital Day 1) demonstrating pulmonary vascular congestion

A chest X-ray was significant for prominent pulmonary vasculature on bilateral lung fields, suggestive of pulmonary edema (Figure 2).

**Figure 3:**
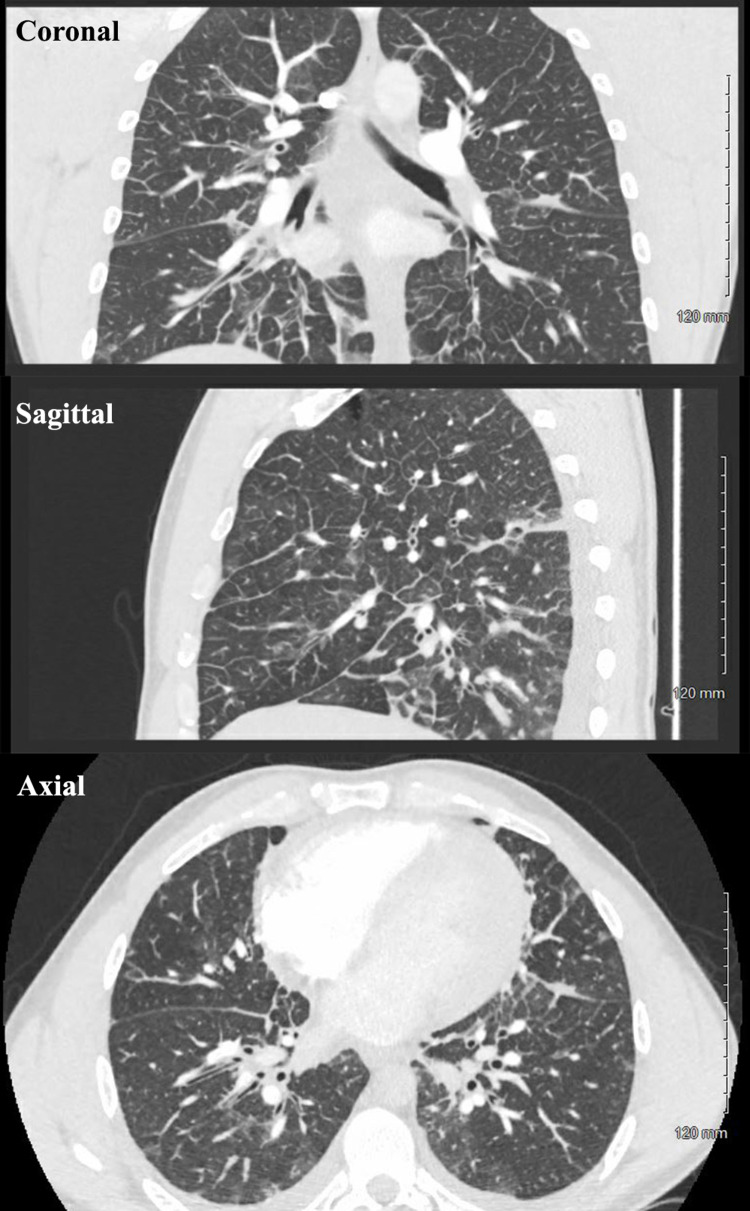
Computed Tomography (CT) Angiography on admission (Hospital day 1) revealing interlobular septal thickening, patchy multifocal ground-glass opacities, small bilateral pleural effusions, and multiple small mediastinal lymph nodes.

The patient scored 4.5 points on the Well’s Criteria for Pulmonary Embolism (PE) and hence was deemed to be of moderate risk for a PE. An elevated D-Dimer level of 1.57 FEU was also noted. A decision was made to perform a Computed tomography (CT) angiogram of the pulmonary vasculature which ruled out a pulmonary embolus. The CT angiogram revealed interlobular septal thickening, patchy multifocal ground-glass opacities, small bilateral pleural effusions, and multiple small mediastinal lymph nodes, and these findings were attributed to pulmonary edema (Figure 3). A diagnosis of congestive heart failure from an acute non-ST elevation myocardial infarction (NSTEMI) was made, and the patient was admitted to the hospital.

Hospital course

**Figure 4:**
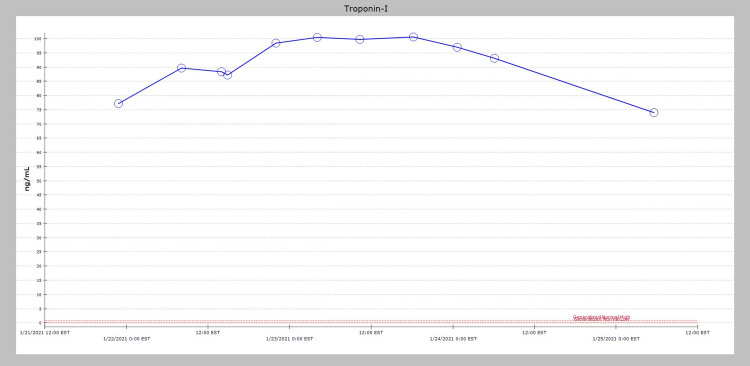
The patient’s troponin I value remained elevated during the hospital stay and peaked at 100.5 ng/mL on Hospital Day 3.

The patient was given aspirin 324 mg and was subsequently started on aspirin 81 mg once daily, atorvastatin 80 mg once daily, carvedilol 6.25 mg two times daily, and a heparin drip for 48 hours as per the hospital protocol for the management of NSTEMI. He was also treated with intravenous furosemide, initially with 20 mg, and escalating doses were later used during the hospitalization. Echocardiogram the next day revealed a left ventricular ejection fraction of 45-50% with hypokinesis of the inferior wall. Coronary artery catheterization revealed no evidence of obstructive coronary artery disease. As per the European Society of Cardiology (ESC) 2013 position statement, the patient satisfied the criteria for clinically suspected myocarditis, and due to timing of use, the SARM RAD-140(testolone) was thought to be the potential etiology [4]. This medication was discontinued in the hospital. Cardiac MRI (CMR) could not be obtained due to COVID restrictions in the hospital. The patient’s troponin I remained elevated during the hospital stay with a peak value approaching 100.5 ng/ml on hospital day three (Figure 4).

The patient responded well to the treatment with diuretics and his clinical status gradually improved during the hospital stay. His repeat echocardiogram on hospital day 7 revealed an improving ventricular function with an ejection fraction of 55-60%. The patient was discharged on hospital day 8 with instructions to follow up at the cardiology clinic in 1-2 weeks.

## Discussion

Shortness of breath on exertion is a common clinical presentation with a myriad of possible diagnoses. A targeted history including a detailed description of the past medical and medication history often provides valuable clues in isolating the etiology. However, in many patients, a definite cause may not be apparent. A systematic approach to investigation is often the key in the timely diagnoses and management of such cases. An analysis of the National Hospital Ambulatory Medical Care Survey from 2005 to 2014 by Hale et al revealed that acute exacerbation of asthma or chronic obstructive pulmonary disease (COPD) is the most common cause of hospitalization for dyspnea on exertion, followed by congestive heart failure [5]. However, congestive heart failure is the most common cause in the elderly, especially in ages 80 and above. A diagnosis of heart failure is exceedingly rare in young individuals with no prior cardiac diseases [6]. When present, heart failure that affects the ventricular function without coronary artery disease is often a sign of non-ischemic cardiac injury, and myocarditis is the most common cause for the same.

Myocarditis should be suspected in a young individual who presents with signs and symptoms consistent with heart failure, without evidence of obstructive coronary artery disease on coronary artery catheterization. Endomyocardial biopsy has long been proposed as the diagnostic gold standard for establishing a diagnosis of acute myocarditis [7]. However, an endomyocardial biopsy is invasive and the sensitivity could be as low as 10-35% [8]. Diagnosis is often clinical, supplemented by laboratory studies. European Society of Cardiology 2013 position statement proposed the criterion to stratify patients with a clinical suspicion of myocarditis and may be helpful for patient selection for endomyocardial biopsy. Cardiac Magnetic Resonance Imaging (CMR) is proposed as a non-invasive alternative and its use is on the rise in the United States [9]. However, availability limits its use. In patients with suspected myocarditis, history and clinical presentation may provide important clues towards a possible etiology. In our patient, the recent self-treatment with the SARM RAD-140 was an important clue.

Testolone RAD-140 is one of several selective androgen receptor modulators, that are currently undergoing testing and research as a potential testosterone alternative. They are promising therapeutics for patients with testosterone deficiency who cannot tolerate anabolic steroids due to their side effect profile. SARMs are currently not approved for use in the US by the Food and Drug Administration (FDA). However, they are currently available for purchase in the US as illegal additions to nutritional supplements and body-building products. They have been added to the World Anti-Doping Agency (WADA) list of prohibited substances, both in and out of competition [10]. The US FDA in 2017 released a warning statement against the use of these products, citing reasons such as “liver damage, increasing the risk of heart attack, and stroke” [11]. As a follow-through, the FDA has since taken many steps to crack down on the illegal distribution of these medications [12]. However, these medications continue to become available to the general public through various means.

The mechanisms with which these medications cause endomyocardial injury remain unclear at this time. Anabolic steroids such as testosterone causing acute myocarditis in patients have been well reported previously [13,14]. Acute myocarditis in relatively young individuals has been traced back to surreptitious testosterone use for bodybuilding [15]. However, to the author’s knowledge, this is the first reported case of a SARM that has been implicated to be causing acute myocarditis.

## Conclusions

Acute myocarditis should be an important differential to consider when patients present with evidence of left ventricular dysfunction and troponin elevation without evidence of coronary artery disease. A wide range of etiologies can cause myocarditis, and careful consideration of history and patient presentation can provide important clues. History of substance abuse and dietary supplementations will need to be carefully considered. Despite the FDA warning, SARM remains available to patients in some capacity and may cause myocarditis and subsequent heart failure in some patients.
